# Healthcare Resource Utilization and Associated Costs among Patients with Advanced Non-Small-Cell Lung Cancer Receiving Chemotherapy or Immunotherapy in Spain: A Single-Center, Real-World, Exploratory Study

**DOI:** 10.3390/cancers16112068

**Published:** 2024-05-30

**Authors:** Jorge Ginés Rubió, Olga Delgado, Angel Callejo, Marta Domínguez, Covadonga Torres

**Affiliations:** 1 Hospital Universitari Son Espases, 07120 Palma de Mallorca, Spain; olga.delgado@ssib.es; 2 OBU Medical Department, AstraZeneca Farmacéutica Spain, 28050 Madrid, Spain; angel.callejo@astrazeneca.com (A.C.); marta.dominguezlopez@astrazeneca.com (M.D.); covadonga.torres@astrazeneca.com (C.T.)

**Keywords:** advanced non-small-cell lung cancer, chemotherapy, immunotherapy, healthcare resource utilization, direct costs, adverse events

## Abstract

**Simple Summary:**

This observational, single-center, retrospective study aimed to describe the real-world (RW) healthcare resource utilization (HCRU) among patients with advanced non-small-cell lung cancer (aNSCLC) who received chemotherapy (CT) or immunotherapy (IT) as first and second lines of treatment. Patients diagnosed with aNSCLC who received at least two cycles of CT or IT as first-line therapy between January 2016 and August 2020 were included. One hundred and seventy-three patients diagnosed with aNSCLC were included in the study; 81.5% and 18.5% received CT and IT as first-line treatment, and 41.5% and 58.5% received CT and IT as second-line treatment. Our results showed that despite the longer treatment exposure with IT, IT use showed a lower average annual cost per patient, which was associated with lower HCRU for both disease and adverse-event management, compared to the use of CT. This finding should be further evaluated in the context of the currently implemented treatment schemes.

**Abstract:**

The objective of this observational, single-center, retrospective study conducted in a Spanish tertiary hospital was to describe the real-world (RW) healthcare resource utilization (HCRU) among patients with advanced non-small-cell lung cancer (aNSCLC) who received chemotherapy (CT) or immunotherapy (IT) as first and second lines of treatment. A total of 173 patients diagnosed with aNSCLC and treated between January 2016 and August 2020 were included. The standardized average costs per patient/year were EUR 40,973.2 and EUR 22,502.4 for first-line CT and IT and EUR 140,601.3 and EUR 20,175.9 for second-line CT and IT, respectively. The average annual costs per patient associated with adverse-event (AE) onset were EUR 29,939.7 and EUR 460.7 for first-line CT and IT and EUR 35,906.4 and EUR 3206.1 for second-line CT and IT, respectively. The costs associated with disease management were EUR 33,178.0 and EUR 22,448.4 for first-line CT and IT and EUR 127,134.2 and EUR 19,663.9 for second-line CT and IT, respectively. In conclusion, IT use showed a lower average annual cost per patient, which was associated with lower HCRU for both disease and AE management, compared to the use of CT. However, these results should be further confirmed in the context of the currently implemented treatment schemes, including the combination of CT with single or dual IT.

## 1. Introduction

Lung cancer is the fourth most frequently diagnosed cancer in Spain in both males and females, following colorectal, prostate, and breast cancers [1,2]. The estimated incidence of new lung cancer cases in Spain in 2020 was 29,638 (21,480 in men and 7708 in women), with a declining trend in males contrasting with a continuous increase in females [3,4]. Lung cancer remains the leading cause of cancer death in Spain for both sexes, with an estimated annual proportion of approximately 20% of all deaths due to cancer [1,4].

Non-small-cell lung cancer (NSCLC) accounts for 85% of all lung cancer cases [5]. Importantly, nearly 70% of NSCLC cases are diagnosed at advanced stages (from IIIB to IV), with locally advanced unresectable or metastatic disease [6,7].

Platinum-based chemotherapy (CT) regimens have long been the standard first-line treatment for advanced non-small-cell lung cancer (aNSCLC) without oncogenic drivers, such as epidermal growth factor receptor (EGFR) mutations and anaplastic lymphoma kinase/oncogene (ALK) or c-ros oncogene 1 (ROS1) rearrangements [8]. However, their response rate has been shown to be limited, ranging between 15% and 30% [9].

Subsequently, the use of a taxane, either alone or in combination with a platinum-based compound, has become the standard second-line therapy for patients with advanced or metastatic NSCLC whose disease progressed upon first-line CT. However, the response rate is often low [10,11].

The variety of treatment options for NSCLC has expanded rapidly over the last 15 years. Molecular biomarkers and cancer immunotherapies have rapidly shifted the traditional treatment paradigms and widened the therapeutic landscape for cancer patients [12]. More specifically, immune checkpoint inhibitors, by targeting and blocking the pathways that control the immune response, such as programmed death-1 (PD-1) and programmed death ligand 1 (PDL-1), have contributed to restoring and maintaining the immune system against cancer cells [13]. Several phase III randomized clinical trials have found that immune checkpoint inhibitors, alone or combined with CT, improve the progression-free and overall survival compared with CT in some NSCLCs, either as second-line therapy for aNSCLC or as first-line therapy in patients with PDL1 expressions on tumor cells ≥ 50% [14]. Likewise, the health-related quality of life (HRQoL) as well as treatment-related adverse-event (AE) severity and incidence seem to improve with immunotherapy (IT) compared with CT [15]. Furthermore, real-world studies have found similar and consistent results, confirming the effectiveness and safety profiles of IT as reported in randomized clinical trials (RCTs) [16]. Consequently, immune checkpoint inhibitors have become a new standard of treatment for aNSCLC and are commonly used in clinical practice [16,17].

Although there is a high amount of healthcare resource utilization (HCRU) and the associated costs derived from aNSCLC management, few real-world studies have assessed the economic impact of HCRU in routine clinical practice [18,19,20,21,22,23]. In fact, a good deal of evidence comes from clinical trials that typically enroll selected patients with aNSCLC who fulfill many exclusion criteria and do not represent the real-world population treated in clinical practice [24]. In addition, RCTs conducted for aNSCLC tend to report significantly lower rates of hospitalization than real-world studies with similar populations and therapies [25]. On the one hand, the high economic burden caused by AEs due to systemic CT compared with IT, in terms of the increased HCRU and hospitalization-associated costs, has rarely been assessed in either clinical trials [26] or real-world studies [27]. On the other hand, due to the great heterogeneity in the access to cancer drugs, the diversity of prescription criteria, and the differences in pricing and reimbursement policies among different EU countries, and even within the same country, the conduction of large, real-world, multicenter studies could be hampered [28,29,30,31].

The main objective of this study was to describe the real-world HCRU among patients with aNSCLC who received CT or IT in a tertiary hospital. Secondarily, this study aimed to assess the costs associated with AEs due to cancer therapy and those related to patient hospitalization.

## 2. Materials and Methods

### 2.1. Study Design

This was an observational, single-center, noninterventional, retrospective study conducted in a Spanish tertiary hospital (Son Espases Hospital, Mallorca). Patients diagnosed with aNSCLC who received at least 2 cycles of CT or IT as first-line therapy between January 2016 [1] and August 2020 [23] were included in this study. The exposure period started when the subject was administered the first cycle (index date). Retrospective data were collected over the observational period (from the index date to the study’s initial date). Only medical resources used in the routine management of these patients were gathered for the analysis. No additional clinical or laboratory assessments or tests were performed.

### 2.2. Ethical Aspects

This study was reviewed and approved by the institutional ethics committee of the Son Espases Hospital (Palma de Mallorca, Spain) and was performed in accordance with all the applicable local regulations on non-interventional studies and/or observational studies. Informed consent was obtained from all enrolled subjects as long as they were alive and attended the consultation.

### 2.3. Study Population and Data Collection

Patients were eligible for the study if they were older than 18 years of age, had been diagnosed with aNSCLC (with histopathological confirmation, either by tissue biopsy or cytology), and had received at least 2 cycles of systemic therapy (CT or IT as first-line treatment after 1 January 2016). In addition, patients must have provided written informed consent, and their complete medical records from the index date to the end of the observational period had to be available. Conversely, patients were excluded if, during the study period, they participated in an oncological clinical trial and/or developed other cancers. In addition, patients with ALK, EGFR, or ROS mutations and those who received treatment with tyrosine kinase inhibitors (TKIs) were excluded from the study selection.

The study data were collected from diverse hospital databases:

1. Sociodemographic data, performed tests, outpatient visits, and AEs were obtained from the Electronic Medical Record (EMR) database using Cerner Millennium^®^ software vs.2018.10.5, a Java and cloud-based automated library;
2. Drug prescriptions, with their respective dates of prescription, doses, and numbers of cycles, were extracted from the pharmacy prescription database based on Farmis-Oncofarm software v.3.0, an ICT that guarantees integrated cancer care;
3. Days of hospitalization due to cancer and/or adverse events were captured from the Hospital Discharge Registry (HDR). This database included main diagnoses, secondary diagnoses, external causes of injury and poisoning, and surgical procedures.

Additionally, further data to evaluate the objectives of the study were obtained by means of an Electronic Case Report Form (eCRF) specifically designed for the study. Each patient selected for the study had a unique and anonymized identifying code.

Finally, the cost analysis was based on cost data from the National Health System eSalud database (Oblikue eSalud), including over 47,000 entries on Spanish health costs, filtered from more than 300 health data sources, retrieved from published articles, books, official health services tariffs of the Spanish Autonomous Communities, and discharge records from National Health System hospitals. All costs were duly updated at the time of the analyses (February 2022) according to the general consumer price index (CPI) interannual rate.

### 2.4. Study Variables

For the primary objective (aNSCLC healthcare resource utilization), the following variables were evaluated: the number of hospitalizations and length of stay due to aNSCLC or adverse events; emergency department (ED) visits related to aNSCLC or to the occurrence of AEs; outpatient visits related to NSCLC or to the occurrence of AEs; pharmacy visits related to aNSCLC; supportive care and/or concomitant (non-cancer-directed) therapies; complementary tests for aNSCLC (imaging tests, biopsy-related procedures, PD-L1 testing, etc.); and the first and second lines of treatment for aNSCLC (including therapies, treatment start and end dates, doses, cycles, and changes/discontinuations).

Among the study’s secondary variables, AEs (whether related or unrelated to antineoplastic and immunosuppressive drugs), the direct costs of all HCRU and those derived from AE management, and sociodemographic and clinical patient characteristics (including age, sex, aNSCLC stage at diagnosis, smoking status, Eastern Cooperative Oncology Group-Performance Status (ECOG-PS), PD-L1 status, comorbidities, and prior treatments for NSCLC) were evaluated.

AEs and toxicities were classified and graded according to the Common Terminology Criteria for Adverse Events (NCI CTCAE version 4.1). In addition, AEs and toxicities were evaluated with the Medical Dictionary for Regulatory Activities (MedDRA) code with the respective system organ classes (SOCs) and preferred terms (PTs).

Regarding antineoplastic or immunosuppressive treatment-related AEs that resulted in hospitalization, ED visits, and outpatient visits, the HDR code (ICD-10 T45.1X5A) was used for identification.

Finally, costs associated with HCRU were calculated by multiplying the natural units of use of each resource by the cost of each resource retrieved from Spanish databases (http://www.oblikue.com/bddcostes/, accessed on 20 January 2021) [32]. The following costs per units of resource were applied for the total cost calculation: hospitalization: 749.95 EUR/day; emergency visit: EUR 296.35; outpatient visit, first visit: EUR 84.67; outpatient visit, consecutive visits: EUR 42.34; and pharmacy visit: EUR 54.43. For the calculation of hospitalizations, the total number of days hospitalized per hospital admission was taken into account. Additionally, the costs for anticancer medication were estimated as described below and included in the total cost calculation. The complementary tests (including analytical and imaging tests) were also calculated (Table S1), although they were not included in the total cost evaluation since these costs could not be assigned to a specific visit category.

### 2.5. Statistical Analysis

It was estimated a priori that the number of patients who could meet the study selection criteria as specified in the study protocol and according to the availability of hospital medical records would be approximately 200. To limit selection bias, all consecutive patients who met the selection criteria were included.

This was an observational and descriptive study without hypothesis testing; hence, no inferential analyses were planned. For the descriptive analysis, all enrolled patients who met the study eligibility criteria were included. The safety set included all enrolled patients with at least one safety assessment.

A univariate descriptive analysis was performed with all the variables collected from the study population. For continuous variables, the mean and standard deviation (SD) and the median and interquartile range (IQR) were calculated. For the categorical variables, the absolute and relative frequencies are presented. Additionally, the 95% confidence interval (CI95%) was calculated for the quantitative variables.

Total HCRU-associated costs were calculated for hospitalizations and visits related to NSCLC disease and AEs, respectively. The cost of each utilized resource is presented either as the sum of visits and hospitalizations or as the mean cost standardized per patient and year. The standardized value was calculated with the following formula: 12 × individual cost/treatment duration (months). Likewise, the cost of any dispensed antineoplastic medication was estimated by multiplying the lowest available cost of the anticancer drug vial by the whole amount of the administered drug during the study follow-up period. With regard to IT, a further 30% discount was applied as an estimation of the difference between the selling price and the reimbursed price. IBM SPSS Statistics 22.0 software was used for the statistical analysis.

## 3. Results

We included 173 patients with a diagnosis of aNSCLC in this study. One hundred and thirty-three patients (67.9%) were male, and the mean age at diagnosis was 63.4 years. The patients’ mean weight was 73.5 kg, 143 (85.2%) had an ECOG-PS of 0–1, 96 (55.5%) presented comorbidities, 168 (97.1%) were either current or former smokers, 151 (88.3%) were diagnosed at stage IV, 53 (30.6%) presented bone metastasis, and 57 (32.9%) had received prior treatment for aNSCLC (Table 1).

Of the study patients, 141 (81.5%) and 32 (18.5%) received CT and IT as first-line treatment, respectively, while for second-line therapy, 41.5% received CT and 58.5% received IT. Supportive and/or concomitant therapies were administered to 136 (78.6%) patients. The baseline clinical profiles of patients treated with CT or IT as first-line therapies were comparable for most characteristics (Table 1). However, a significantly higher frequency of ECOG-PS grade 2 was observed in the patients treated with CT compared with those treated with IT (15.4% vs. 6.3%). Likewise, there were significantly less patients previously treated with either surgery plus CT (8.6% vs. 18.2%) or radiotherapy plus CT (17.1% vs. 63.6%) in the CT group compared with the IT group (Table 1).

**Figure 1:**
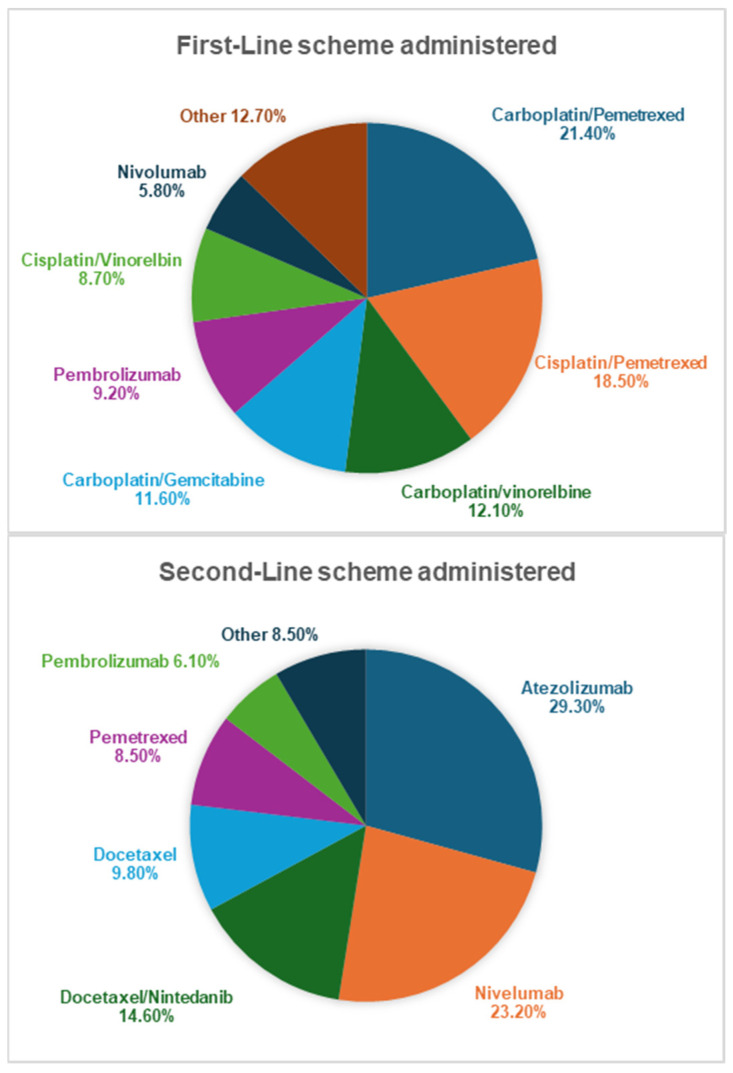
Treatments administered in first and second lines of anticancer therapy.

The mean (SD) first-line and second-line treatment durations as well as the reductions and interruptions in the therapies are presented in Table 2. IT was associated with fewer treatment reductions and interruptions despite the longer exposure time. Carboplatin/pemetrexed (21.4%) and cisplatin/pemetrexed (18.5%) were the most commonly administered drugs in the first-line scheme, whereas atezolizumab (29.3%), nivolumab (23.2%), and the combination of docetaxel–nintedanib (14.6%) were the most frequently administered drugs within the second line. The respective first- and second-line CT and IT drugs are shown in Figure 1.

Of the patients, 169 (97.7%) reported at least one AE, and 83.5% of the AEs were related to CT administration, while 16.5% were associated with IT. The most frequent adverse events related to CT in the first-line setting were anemia (68.1%), asthenia (62.4%), and neutropenia (31.2%). Notably, the neutropenia severity was reported as grade 3 and 4 in 56.8% of the cases. Likewise, the most commonly reported AEs with second-line CT were asthenia (39.4%), anemia (30.3%), and diarrhea (24.2%). Conversely, the incidence of AEs related to IT in either of the therapy lines was much lower. The most frequently reported AEs were asthenia with 37.5% and 31.3% for first- and second-line therapy, respectively, and diarrhea (18.6%) for first-line therapy (Table 3). In addition, the most frequent immune-mediated adverse events with first-line IT were pneumonitis (9.4%), hypophysitis (6.3%), hypothyroidism (6.3%), arthralgia (6.3%), nephritis (6.3%), and erythema (6.3%) (Table 3).

With regard to the utilization of healthcare resources (HCRU), the study patients were hospitalized within a frequency range from 43.8% (first-line IT) to 58.2% (second-line CT), with an average (SD) hospital stay of 21 (23.8) days. The respective numbers of treatment days per therapy and line of treatment (LOT) are displayed in Table 4. Moreover, from 39.6% to 69.7% of the patients visited the ED, and almost 100% of the study population had at least one outpatient visit. Interestingly, hospitalizations, ED visits, and pharmacy visits were more frequent in the subgroup of patients treated with CT than in those who received IT regardless of the LOT (see Table 4). Differences in HCRU seemed to be even more marked when compared with those utilizing HCR related to AE onset (Table 4).

Regarding the total direct costs associated with HCRU during the study period, the standardized average costs per patient/year, including the cost of the administered anticancer medication, were EUR 46,282.4 and EUR 38,900.5 for first-line CT and IT and EUR 156,610.6 and EUR 35,778.5 for second-line CT and IT, respectively (Table 5). Furthermore, the average annual costs per patient of visits and hospitalizations associated with cancer treatment-related AE onset and the consequent management were EUR 29,939.7 and EUR 460.7 for first-line CT and IT and EUR 35,906.4 and EUR 3206.1 for second-line CT and IT. The AE-associated costs derived from the specific HCRUs are displayed in Table 4. Likewise, the average annual costs per patient of the visits and hospitalizations associated with disease management were EUR 33,178.0 and EUR 22,448.4 for first-line CT and IT and EUR 127,134.2 and EUR 19,663.9 for second-line CT and IT, respectively (Table 5). Although the average annual antineoplastic drug cost per patient was lower for CT (EUR 5309.1) than for IT (EUR 16,398.1) for first-line treatment, CT resulted in higher costs during second-line treatment, with annual drug costs per patient of EUR 16,009.3 and EUR 15,602.5 for CT and IT, respectively (Table 5).

## 4. Discussion

Our results indicate that patients with aNSCLC who received CT or IT were associated with high HCRU and related costs. These costs were higher in the subgroup of patients who received CT than in those who were treated with IT in both the first- and second-line settings, although the cost of CT for first-line treatment was lower than the cost of IT.

Overall, our findings regarding HCRU associated with the first and second lines of anticancer therapy are consistent with those reported by three large real-world European multinational studies conducted in the preimmunotherapy era, although no comparisons between the HCRU and costs associated with a diverse line of therapies were performed in these studies [19,22,33]. Hospitalizations related to first-line anticancer systemic therapy ranged between 42% [33] and 55% [19] and are consistent with our findings (from 44% to 58%). However, the average hospital length of stay in our study (21 days) was longer than those reported in the aforementioned studies (ranging from 8 to 13 days) [19,22], which may partly explain the difference in the total costs associated with HRCU between previous studies and this study.

Regardless of the line of therapy, a higher proportion of patients treated with CT were admitted to the hospital, visited the ED, and consumed pharmacy resources. Moreover, patients who received CT in both lines of therapy reported a higher incidence and severity of treatment-related AEs. Importantly, the HCRU associated with AEs was greater in the CT subgroup of patients. Thus, the largest differences in the HCRU between CT and IT were in the AE-related hospitalizations and visits to the ED, which also translated into an enormous difference in costs in the first-line and, especially, second-line settings. Therefore, treatment-emergent AEs were the major driver of the HCRU and costs in our study. Similar results have been reported in a large, real-world, retrospective study using a US administrative claims database: AEs were less frequent and the total AE costs were significantly lower in patients treated with IT than in those treated with CT either alone or in combination with IT [27]. Furthermore, according to the study authors, the higher AE costs seemed to have been mainly driven by hospitalizations and ED visits [27], which is in line with our results. We observed a higher frequency of hospitalizations and ED visits as well as visits to the pharmacy in the CT-treated subgroup of patients, which contrasts with the reported frequencies of outpatient visits between CT- and IT-treated patients in both LOTs.

To some extent, our results support the lower HCRU and associated direct costs derived from the use of IT as a first choice of anticancer treatment for patients with aNSCLC, and they complement findings from other studies that corroborate its cost-effectiveness [34].

To the best of our knowledge, this study is the first medical-record-review, real-world, retrospective study to compare the HCRU and costs associated with CT and IT administered as first- and second-line anticancer therapy to patients diagnosed with aNSCLC. One strength of our study is that, despite the fact that it was conducted in a single center, the evaluated data did not come from all-payer claims databases but were instead collected from various reliable hospital databases, such as the EMR database, the hospital pharmacy prescription database, and the HDR, as explained above. However, some study limitations must be acknowledged. We observed a low number of IT-treated patients in both the first and second lines, which prevents the establishment of a definite conclusion. In addition, because this was an observational, retrospective, chart-review study, missing or incorrectly recorded data could not be discarded. Specifically, the HCRU associated with AEs, particularly the number of hospitalizations, could not be causally established; therefore, we cannot exclude an underestimation of the HCRU. The cost of second-line anticancer medication could be explained, at least in part, by the relatively high proportion of patients treated with the combination of docetaxel–nintedanib. Furthermore, anticancer drug-associated costs and expenses due to the complementary tests were not included in the total direct costs. Although this was not the goal of our study, we must state that there were further related direct health costs that included, among others, medication, healthcare material, preparation, distribution, administration, adverse-effect management, and hospitalization costs. Furthermore, it is also necessary to consider the direct non-health costs assumed by patients or relatives, such as travel costs, resources consumed by the patient and the family, the use of social services, etc. Moreover, we also have to acknowledge the indirect costs, a consequence of the lack of productivity—whether paid or unpaid—of the patients and/or their caregivers. Therefore, our study did not encompass the whole picture of the HCRU and costs associated with CT and IT. Importantly, the present study did not cover all the currently accepted treatment strategies, and we did not assess the HCRU and costs associated with the combination of CT and IT. Another limitation of the study is that we did not compare the costs for CT and IT adjusting for clinical variables that could differ between these treatment groups. Furthermore, although the clinical profiles of both groups were generally comparable, some baseline clinical characteristics diverge between patients treated with either CT or IT as first-line therapy, such as their ECOG-PSs and former medical histories of CT combined with surgery or radiotherapy.

At the time that our study was conducted, the most commonly implemented treatment schemes for aNSCLC were either CT or IT as monotherapies. This paradigm has changed significantly in recent years [35], and new IT agents that target different pathways in combination with CT have been incorporated into treatment algorithms as a result of the positive outcomes of many landmark studies [36,37,38,39] and following subsequent FDA approval [40,41]. Hence, several therapy options are now available that combine classical platinum-based CT with either a single or dual IT agent [35,42]. Consequently, considering that the above results reflect the experience from a single center, and that the landscape of the NSCLC treatment has substantially changed over the past few years, we cannot assure that the study results completely capture the current clinical practices, such as the combination of CT and IT, and, hence, any generalization should be cautiously made [28,43]. Currently, upfront programmed death ligand 1 (PD-L1) expression testing before first-line therapy is also a recommendation (regardless of the histology) for patients with advanced or metastatic NSCLC, in order to assess how immune checkpoint inhibitors could be used if no actionable molecular biomarkers are identified [44,45]. Treatment options for advanced or metastatic NSCLC without actionable molecular biomarkers are stratified by the PD-L1 level and include systemic therapy options such as immunotherapy with or without chemotherapy [46]. Similarly, with the increased use of the combination of immunotherapy and chemotherapy or immunotherapy monotherapy as a first-line treatment, the use of second-line immunotherapy will decrease, relegating patients who have not previously received PD-1/PD-L1 inhibitors [47]. Future real-world prospective studies with large sample sizes are needed to assess the comparative cost-effectiveness of the novel immunotherapy and target therapy for NSCLC.

## 5. Conclusions

In a real-world setting, in both the first and second lines of treatment for aNSCLC, and despite the longer treatment exposure with IT and its direct associated cost, IT use showed a lower average annual cost per patient, associated with lower HCRU for the management of both the disease itself and treatment-emergent AEs, than the use of CT. However, these results should be further confirmed within the presently implemented treatment schemes, including the combination of CT with single or dual IT. This will help health professionals make decisions about incorporating new cost-effective therapy approaches into NSCLC treatment management.

## Figures and Tables

**Table 1:** Sociodemographic and clinical characteristics.

	First-Line Chemotherapy		First-Line Immunotherapy		Total		*p*-Value
Characteristics	N		N		N		
Age, years. Mean (SD)	141	65.0 (7.8)	32	63.1 (9.4)	173	63.4 (9.1)	0.288
Gender (male), n (%)	141	109 (77.3)	32	24 (75.0)	173	133 (76.9)	0.780
Weight, kg. Mean (SD)	141	73.6 (15.5)	32	72.7 (15.5)	173	3.5 (15.4)	0.765
ECOG-PS, n (%)	136 *		32		168 *		0.028
0		36 (26.5)		14 (43.8)		50 (29.8)	
1		77 (56.6)		16 (50.0)		93 (55.4)	
2		21 (15.4)		2 (6.3)		23 (13.7)	
3		2 (1.5)		0 (0.0)		2 (1.2)	
Smoking status, n (%)	141		32		173		0.142
Nonsmoker		4 (2.8)		1 (3.1)		5 (2.9)	
Former smoker		62 (44.0)		19 (59.4)		81 (46.8)	
Current smoker		75 (53.2)		12 (37.5)		87 (50.3)	
Stage at NSCLC diagnosis (locally advanced or metastatic), n (%)	139 *		32		171 *		NA **
IIIA		2 (1.4)		0 (0.0)		2 (1.2)	
IIIB		10 (7.2)		3 (9.4)		13 (7.6)	
IIIC		5 (3.6)		0 (0.0)		5 (2.9)	
IVA		82 (59.0)		23 (71.9)		105 (61.4)	
IVB		40 (28.8)		6 (18.8)		46 (26.9)	
Metastatic locations, >10%, n (%)	141		32		173		
Bone		44 (31.2)		9 (28.1)		53 (30.6)	0.733
Pleural		27 (19.1)		6 (18.8)		33 (19.1)	0.959
Lung		29 (20.6)		4 (12.5)		33 (19.1)	0.294
Visceral		21 (14.9)		7 (21.9)		28 (16.2)	0.333
CNS		17 (12.1)		3 (9.4)		20 (11.6)	0.668
Suprarenal		17 (12.1)		3 (9.4)		20 (11.6)	0.668
Tumor histopathology, n (%)	141		32		173		0.385
Adenocarcinoma		97 (68.8)		20 (62.5)		117 (67.6)	
Squamous cell carcinoma		29 (20.6)		12 (37.5)		41 (23.7)	
Other		15 (10.6)		0 (0.0)		15 (8.7)	
PDL-1 expression, n (%)	85 *	34 (40.0)	26 *	20 (76.9)	111 *	54 (48.6)	0.001
Comorbidities (yes), n (%)	141	78 (55.3)	32	18 (56.3)	173	96 (55.5)	0.924
Charlson Comorbidity Index score. Mean (SD)	78 *	2.3 (1.8)	18 *	1.6 (0.7)	96 *	2.2 (1.6)	0.086
Prior treatments for NSCLC, n (%)	35 *		22 *		57 *		
Surgery		12 (34.3)		6 (27.3)		18 (31.6)	0.087
Radiotherapy		17 (48.6)		6 (27.3)		23 (40.4)	0.385
Surgery + CT		3 (8.6)		4 (18.2)		7 (12.3)	0.023
Radiotherapy + CT		6 (17.1)		14 (63.6)		20 (35.1)	<0.01
Other anticancer treatment		5 (14.3)		4 (18.2)		9 (15.8)	0.062
Time since diagnosis of advanced/metastatic NSCLC, months. Mean (SD)	141	28.4 (14.5)	32	20.9 (13.3)	173	27.0 (14.6)	0.008

* Number of patients inferior to the study population (N = 173) due to missing data. ** Not calculated because the frequency of more than 25% of the cells is less than 5. CNS: central nervous system; ECOG-PS: Eastern Cooperative Group Performance Status; N: number of evaluable patients; NSCLC: non-small-cell lung cancer; kg: kilogram; SD: standard deviation

**Table 2:** Durations and modifications of therapies for aNSCLC.

Therapies for aNSCLC	First-Line Chemotherapy	First-Line Immunotherapy	Second-Line Chemotherapy	Second-Line Immunotherapy
Treatment, n (%)	141 (81.5)	32 (18.5)	34 (58.5)	48 (41.5)
Treatment duration, months. Mean (SD)	3.4 (2.8)	7.6 (7.6)	1.9 (2.6)	5.9 (7.8)
Treatment reductions, n (%)	56 (39.7)	1 (3.1)	9 (26.5)	0
Treatment interruptions, n (%)	65 (46.1)	14 (43.8)	14 (41.2)	9 (18.8)

aNSCLC: advanced non-small-cell lung cancer; SD: standard deviation.

**Table 3:** Adverse-event incidence and severity per aNSCLC therapy and line of treatment (≥5%).

				Grade													
				1		2		3		4		5		NA		TOTAL	
LOT	T	SOC	PT	N	%	N	%	N	%	N	%	N	%	N	%	N	%
First line (N = 173)	CT (N = 141)	Blood and lymphatic system disorders	Anemia	26	18.4	48	34.0	21	14.9	1	0.7	0	0.0	0	0.0	96	68.1
			Febrile neutropenia	0	0.0	0	0.0	2	1.4	5	3.5	0	0.0	0	0.0	7	5.0
			Neutropenia	3	2.1	16	11.3	14	9.9	11	7.8	0	0.0	0	0.0	44	31.2
			Thrombocytopenia	3	2.1	3	2.1	1	0.7	1	0.7	0	0.0	0	0.0	8	5.7
		Gastrointestinal disorders	Diarrhea	12	8.5	3	2.1	2	1.4	0	0.0	0	0.0	0	0.0	17	12.1
			Nausea	26	18.4	12	8.5	2	1.4	0	0.0	0	0.0	1	0.7	41	29.1
			Vomiting	7	5.0	3	2.1	2	1.4	0	0.0	0	0.0	0	0.0	12	8.5
		General disorders and administration site conditions	Asthenia	31	22.0	26	18.4	21	14.9	2	1.4	0	0.0	8	5.7	88	62.4
			Mucosal inflammation	9	6.4	2	1.4	2	1.4	1	0.7	0	0.0	0	0.0	14	9.9
		Investigations	Platelet count decrease	10	7.1	6	4.3	3	2.1	7	5.0	0	0.0	0	0.0	26	18.4
		Metabolism and nutrition disorders	Decreased appetite	7	5.0	13	9.2	2	1.4	0	0.0	0	0.0	11	7.8	33	23.4
		Nervous system disorders	Dysgeusia	5	3.5	1	0.7	0	0.0	0	0.0	0	0.0	11	7.8	17	12.1
		Respiratory, thoracic, and mediastinal disorders	Pulmonary embolism	1	0.7	0	0.0	0	0.0	0	0.0	0	0.0	13	9.2	14	9.9
	IT (N = 32)	Blood and lymphatic system disorders	Anemia	2	6.3	2	6.3	1	3.1	0	0.0	0	0.0	0	0.0	5	15.6
			Neutropenia	1	3.1	1	3.1	0	0.0	0	0.0	0	0.0	0	0.0	2	6.3
		Endocrine disorders	Hypophysitis	0	0.0	0	0.0	0	0.0	0	0.0	0	0.0	2	6.3	2	6.3
		Gastrointestinal disorders	Diarrhea	2	6.3	1	3.1	1	3.1	1	3.1	0	0.0	0	0.0	5	15.6
			Nausea	2	6.3	0	0.0	0	0.0	0	0.0	0	0.0	0	0.0	2	6.3
		General disorders and administration site conditions	Asthenia	7	21.9	3	9.4	1	3.1	0	0.0	0	0.0	1	3.1	12	37.5
		Metabolism and nutrition disorders	Decreased appetite	2	6.3	1	3.1	0	0.0	0	0.0	0	0.0	0	0.0	3	9.4
			Hypomagnesaemia	0	0.0	0	0.0	0	0.0	0	0.0	0	0.0	3	9.4	3	9.4
			Hypothyroidism	1	3.1	0	0.0	0	0.0	0	0.0	0	0.0	1	3.1	2	6.3
		Musculoskeletal and connective tissue disorders	Arthralgia	1	3.1	0	0.0	0	0.0	0	0.0	0	0.0	1	3.1	2	6.3
		Nervous system disorders	Dysgeusia	2	6.3	0	0.0	1	3.1	0	0.0	0	0.0	0	0.0	3	9.4
		Renal and urinary disorders	Nephritis	1	3.1	1	3.1	0	0.0	0	0.0	0	0.0	0	0.0	2	6.3
		Respiratory, thoracic, and mediastinal disorders	Pneumonitis	0	0.0	1	3.1	0	0.0	0	0.0	0	0.0	2	6.3	3	9.4
		Skin and subcutaneous tissue disorders	Erythema	1	3.1	1	3.1	0	0.0	0	0.0	0	0.0	0	0.0	2	6.3
2nd line (N = 82)	CT (N = 33)	Blood and lymphatic system disorders	Anemia	0	0.0	8	24.2	2	6.1	0	0.0	0	0.0	0	0.0	10	30.3
			Febrile neutropenia	0	0.0	0	0.0	1	3.0	1	3.0	0	0.0	0	0.0	2	6.1
			Neutropenia	1	3.0	1	3.0	1	3.0	2	6.1	0	0.0	0	0.0	5	15.2
		Gastrointestinal disorders	Constipation	0	0.0	0	0.0	0	0.0	1	3.0	0	0.0	1	3.0	2	6.1
			Diarrhea	2	6.1	4	12.1	0	0.0	0	0.0	0	0.0	2	6.1	8	24.2
			Nausea	1	3.0	1	3.0	0	0.0	0	0.0	0	0.0	1	3.0	3	9.1
			Vomiting	0	0.0	0	0.0	0	0.0	0	0.0	0	0.0	2	6.1	2	6.1
		General disorders and administration site conditions	Mucosal inflammation	2	6.1	2	6.1	0	0.0	0	0.0	0	0.0	1	3.0	5	15.2
		Investigations	Platelet count decrease	0	0.0	0	0.0	0	0.0	2	6.1	0	0.0	0	0.0	2	6.1
		Metabolism and nutrition disorders	Decreased appetite	2	6.1	1	3.0	2	6.1	0	0.0	0	0.0	1	3.0	6	18.2
		Musculoskeletal and connective tissue disorders	Musculoskeletal pain	0	0.0	0	0.0	0	0.0	0	0.0	0	0.0	2	6.1	2	6.1
		Skin and subcutaneous tissue disorders	Alopecia	0	0.0	0	0.0	0	0.0	0	0.0	0	0.0	3	9.1	3	9.1
			Rash	0	0.0	2	6.1	0	0.0	0	0.0	0	0.0	1	3.0	3	9.1
	IT (N = 48)	Blood and lymphatic system disorders	Anemia	1	2.1	2	4.2	2	4.2	0	0.0	0	0.0	0	0.0	5	10.4
		Gastrointestinal disorders	Diarrhea	1	2.1	1	2.1	1	2.1	0	0.0	0	0.0	0	0.0	3	6.3
		General disorders and administration site conditions	Asthenia	10	20.8	5	10.4	0	0.0	0	0.0	0	0.0	0	0.0	15	31.3
		Hepatobiliary disorders	Hepatitis	0	0.0	1	2.1	1	2.1	0	0.0	0	0.0	1	2.1	3	6.3
		Metabolism and nutrition disorders	Hypomagnesaemia	0	0.0	0	0.0	0	0.0	0	0.0	0	0.0	3	6.3	3	6.3
			Hypothyroidism	2	4.2	0	0.0	0	0.0	0	0.0	0	0.0	1	2.1	3	6.3
		Musculoskeletal and connective tissue disorders	Arthralgia	2	4.2	0	0.0	0	0.0	0	0.0	0	0.0	1	2.1	3	6.3

LOT: line of therapy; T: therapy; SOC: system organ class; PT: preferred term.

**Table 4:** Healthcare resource utilization per aNSCLC therapy and line of treatment.

Healthcare Resources/Year per Patient	First-Line Chemotherapy N = 141	First-Line Immunotherapy N = 32	Second-Line Chemotherapy N = 33	Second-Line Immunotherapy N = 48
Hospitalizations *, n (%)	82 (58.2)	14 (43.8)	19 (57.6)	22 (45.8)
N of days per hospitalization *. Mean (SD)	8.5 (6.0)	9.9 (6.8)	10.9 (6.4)	7.6 (5.2)
Hospitalizations related to aNSCLC, n (%)	68 (48.2)	13 (40.6)	14 (42.4)	21 (43.8)
N of days per hospitalization related to aNSCLC. Mean (SD)	8.9 (6.6)	10.4 (6.7)	11.2 (6.1)	7.8 (5.7)
Hospitalizations related to AEs, n (%)	21 (14.9)	1 (3.1)	6 (18.2)	4 (8.3)
N of days per Hospitalization related to AEs. Mean (SD)	10.3 (6.9)	3,0	10.3 (6.9)	7.8 (2.6)
ED visits *, n (%)	76 (53.9)	16 (50.0)	22 (66.7)	19 (39.6)
ED visits related to aNSCLC, n (%)	66 (46.8)	16 (50.0)	15 (45.5)	17 (35.4)
ED visits related to AEs, n (%)	16 (11.3)	0	7 (21.2)	2 (4.2)
Outpatient visits *, n (%)	141 (100)	32 (100)	32 (97)	48 (100)
Outpatient visits related to aNSCLC, n (%)	141 (100)	32 (100)	32 (97)	48 (100)
Outpatient visits related to AEs, n (%)	20 (14.2)	4 (12.5)	7 (21.2)	4 (8.3)
Pharmacy visits related to aNSCLC, n (%)	76 (53.9)	4 (12.5)	17 (51.5)	1 (2.1)

AE: adverse event; aNSCLC: advanced non-small-cell lung cancer; ED: emergency department. * Related to aNSCLC or AEs.

**Table 5:** Direct healthcare costs per aNSCLC therapy and line of treatment.

Mean Cost Standardized per Patient and Year	First-Line Chemotherapy	First-Line Immunotherapy	Second-Line Chemotherapy	Second-Line Immunotherapy
Hospitalization	59,953.8€	43,346.5€	224,195.7€	38,345.1€
ED visits	3051.6€	1649.0€	5491.0€	1342.8€
Outpatient visits	3758.5€	2600.8€	4884.4€	2701.2€
Pharmacy visits	1304.7€	903.9€	1184.3€	29.6€
Mean costs of hospitalizations and visits associated with AEs	29,839.7€	460.7€	35,906.4€	3206.1€
Mean costs of hospitalizations and visits associated with aNSCLC	33,178.0€	22,448.4€	127,134.2€	19,663.9€
Mean total visit costs	40,973.2€	22,502.4€	140,601.3€	20,175.9€
Mean cost of anticancer medication (immunotherapy 30% discount)	5309.1€	16,398.1€	16,009.3€	15,602.5€
Mean total costs (treatment costs + visits cost)	46,282.4€	38,900.5€	156,610.6€	35,778.5€

AE: adverse event; aNSCLC: advanced non-small-cell lung cancer; ED: emergency department. Total healthcare-associated costs were calculated for hospitalizations and visits related to NSCLC disease and adverse events, respectively. The cost of each utilized resource is presented either as the sum of visits and hospitalizations or as the mean cost standardized per patient and year. The standardized value was calculated with the following formula: 12 × individual cost/treatment duration (months).

## Data Availability

The data that support the findings of this study are available from the corresponding author upon reasonable request.

## Supplementary Materials

The following supporting information can be downloaded at: https://www.mdpi.com/article/10.3390/cancers16112068/s1, Table S1. Costs of complementary tests *.
