# Supplementary material for: Healthcare Resource Utilization and Associated Costs among Patients with Advanced Non-Small-Cell Lung Cancer Receiving Chemotherapy or Immunotherapy in Spain: A Single-Center, Real-World, Exploratory Study
Source: Cancers (Basel). 2024 May 30;16(11):2068. doi: 10.3390/cancers16112068 (PMC11171292; doi:10.3390/cancers16112068)
Supplement: Supplementary file 1 [file cancers-16-02068-s001.zip › cancers-2997577-supplementary.pdf]

Table S1. Costs of complementary tests *.

TEST	Unit cost (€)
Hematology and biochemistry	73.58
Urinalysis	7.33
Coagulation test	14.36
Hormone test	1160.98
Biopsy	52.42
PD-L1 test	Not available
Imaging tests:<br>CT scan	207.65
Radiograph	58.46
PET scan	486.99
MRI	507.02
Ultrasound	5098.46
Angio ACT	236.81
Bone gammagraphy	150.12
Bone gammagraphy transthoracic	195.55
Fibrobroncoscopy	145.15
Echocardiogram	195.55
Abdominal echography	82.66
Eco-doppler	128.02
Shoulder echography	82.66
Urinary tract echography	82.66
Abdominal-pelvic echography	120.96
Pulmonary artery ACT	376.99
Thyroid gammagraphy	44.61
Gastroscopy	442.51
Bronchoscopy	174.38
Soft tissue echography (armpit):	82.66
Bone SPECT-CT	502.99
Cervical eco-doppler	120.96
Abdominal-renal echography	82.66
Thoracic echography	82.66
Coronary angiography	1468
Other tests:<br>Glycosylated hemoglobin	9.07
Ascites fluid culture cost	84.01
PSA	55.44
HIV serology	177.41
Hemoculture	22.18
Pleural fluid culture	84.01
NT-PRO-BNP	88.25
Swab culture	12.10
Stool culture	96.77
Exudate swab	12.10
Treponema pallidum serology	20.05
Sputum culture	23.18
Synovial fluid culture	84.01
B12 vitamin	21.17

Electrocardiogram	59.47
CEA	8.06
Copper	18.14
Selenium	19.15
Zinc	9.07
Coombs test	32.26
Hepatitis B serology	55.44
Hepatitis A serology	55.44
Cytogenetic study	115.92
Bronchial aspirate culture	36.29
Bone marrow aspiration	454.03
Catheter culture	17.14
HER2-CEP determination	406.22
CRP	282.24
Urinary antigens study	51.44
Vitamin D	23.18
Calcium	6.05
Troponin	28.22
Urine sediment	8.06
PEG	925.34
Liquid culture	26.00
TPO antibodies	10.75
Vaginal culture	28.00

PD-L1: Programmed Death-ligand 1; CT: Computed Tomography; ACT: Axial Computed Tomography; SPECT-CT: Photon Emission Computed Tomography-Computed Tomography; PET: Positron Emission Tomography; MRI: Magnetic Resonance Imaging; PSA: Prostate-specific Antigen; HIV: Human Immunodeficiency Virus; NT-PRO-BNP: N-Terminal-pro-Brain Natriuretic Peptide; CEA: Cancer Embryonic Antigen; HER2-CEP: Human Epidermal Growth Factor Receptor 2 - Chromosome Enumeration Probe 17; CRP: C-Reactive Protein; PEG: Percutaneous Endoscopic Gastrostomy; TPO: Thyroperoxidase.
*Source of Spanish health care costs database: eSalud platform set up by Oblikue Consulting.
